# Educational inequalities in metabolic syndrome prevalence, timing, and duration amongst adults over the life course: a microsimulation analysis based on the lifelines cohort study

**DOI:** 10.1186/s12966-023-01495-1

**Published:** 2023-09-04

**Authors:** Liza A. Hoveling, Alexander Lepe, Michael Boissonneault, Joop A. A. de Beer, Nynke Smidt, Marlou L. A. de Kroon, Aart C. Liefbroer

**Affiliations:** 1 Department of Epidemiology, University of Groningen, University Medical Center Groningen, PO Box 30.001, Groningen, 9700 RB The Netherlands; 2 Department of Health Sciences, Community and Occupational Medicine, University of Groningen, University Medical Center Groningen, PO Box 30.001, Groningen, 9700 RB The Netherlands; 3 Netherlands Interdisciplinary Demographic Institute (NIDI)-KNAW, University of Groningen, PO Box 11650, The Hague, 2502 AR The Netherlands; 4 Environment and Health, Department of Public Health and Primary Care, KU Leuven, Leuven, Belgium; 5 Department of Sociology, Vrije Universiteit Amsterdam, De Boelelaan 1081, Amsterdam, 1081 HV The Netherlands

**Keywords:** Metabolic syndrome, Education, Smoking, Alcohol drinking, Diet, Health literacy, Microsimulation

## Abstract

**Background:**

Educational inequalities in metabolic syndrome (MetS) are a growing public health concern. Intervening on modifiable factors may help reduce these inequalities, but there is a need for evidence on the long-term impact of intervening on these factors. Thus, we simulate the development of educational inequalities in MetS across the life course and assess the impact of intervening on the modifiable factors that contribute to these inequalities.

**Methods:**

We used data from the prospective multigenerational Dutch Lifelines Cohort Study to estimate the required input for a continuous-time microsimulation. The microsimulation projects the development of educational inequalities in MetS between ages 18 and 65, and assesses the potential benefit of intervening on smoking, alcohol use, diet quality, and health literacy.

**Findings:**

The likelihood of ever experiencing MetS between ages 18 and 65 varies from 32.5% among high educated women to 71.5% among low educated men. On average, 27.6% more individuals with low education will ever experience MetS between ages 18 and 65 compared to those with high education. Additionally, individuals with low education generally will develop MetS 2.3 years earlier, and will spend an extra 2.6 years with MetS, compared to individuals with high education. Changes to smoking behaviours in individuals with low education produced the largest effect; it would reduce inequalities in prevalence, timing and duration by an average of 7.5%, 9.5%, and 6.9%, respectively.

**Conclusions:**

Interventions targeting the modifiable factors included in this study, especially smoking, could help reduce the estimated educational inequalities in MetS over the life course.

**Supplementary Information:**

The online version contains supplementary material available at 10.1186/s12966-023-01495-1.

## Introduction

Educational health inequalities amongst adults are a significant public health issue, and are expected to widen [1]. Individuals with low education are more likely to develop adverse health conditions [2], do so earlier in life, and have a lower healthy life expectancy than individuals with high education [3]. Reducing these educational inequalities would benefit the well-being of both individuals and society [4].

Studies have found large educational gradients in metabolic syndrome (MetS) in both children and adults [2, 5, 6], but evidence on the development of these inequalities over the life course is lacking. MetS is a cluster of interrelated risk factors, which increases the risk of cardiovascular disease (CVD) and type 2 diabetes [7]. MetS is defined as the co-occurrence of at least three of the following factors: abdominal obesity, elevated triglyceride levels, low high-density lipoprotein cholesterol levels, elevated blood pressure, and elevated fasting glucose levels [8]. With a prevalence of 10–30% in European adults, MetS is a serious public health problem and due to increasing trends in overweight and obesity in the coming years, even more individuals are expected to develop MetS [9].Cross-sectional studies have shown that individuals with low educational attainment are more likely to have MetS [5]. Additionally, prospective studies have shown that individuals with low education have a higher risk to develop MetS, and are also less likely to recover from MetS [2, 10]. However, there is a lack of evidence on the development of educational inequalities in MetS over the life course, which is important as cardiometabolic risk has been shown to vary over the life course and these trajectories differ according to sex [7].

Given the seriousness of educational inequalities in MetS, examining to what extent intervening on modifiable factors could reduce these inequalities is important as well. Studies show that individuals with high education have healthier behaviours [11] resulting in a lower risk of developing MetS and a higher likelihood of recovering from MetS than those with less education [2, 10]. For example, studies have shown that individuals with low education smoke more often, have more unhealthy diets, and have lower health literacy levels compared to their counterparts with high education [12–15]. In turn, these factors are associated with higher risk to develop MetS [16–18] and lower likelihood of recovering from MetS [19, 20]. In other words, modifiable factors partially explain educational inequalities in MetS development and recovery. However, what the consequences of changing these modifiable factors are at the population level in terms of reducing educational inequalities is unknown. Microsimulation offers opportunities to examine the extent to which – at the population level – changes in modifiable risk factors could reduce educational inequalities in the prevalence, timing, and duration of MetS over the entire life course.

Our aims are to (1) simulate, based on empirical data from a large-scale population-based longitudinal study, the development of MetS across the life course, and (2) estimate the impact of intervening on modifiable factors that contribute to educational inequalities in MetS development over the life course.

## Methods

We use microsimulation to construct individual life courses for a synthetic cohort. Individuals within this cohort are allowed to transition between two states, i.e. having and not having MetS. Transition rates are based on MetS incidence and recovery rates derived from empirical data from the Lifelines Cohort Study [21]. The individual life courses are then characterized by the sequence of states and the amount of time spent in each state over time. In our specific application, we proceed in two steps. First, we simulate life courses stratified by sex and education. These life courses are then used to estimate a set of population parameters. Second, given that individuals with low and high education levels differ across various modifiable risk factors, we conduct a counterfactual analysis. The counterfactual analysis allows us to consider what the educational inequalities in MetS would look like if the distribution of the modifiable factors in individuals with low education was the same as in individuals with high education.

### Simulating educational inequalities in MetS across the life course

The estimation of the microsimulation model was performed using the MicSim package in R version 4.0.2 [22]. We use the micSim function to create a continuous-time microsimulation, which simulates individual life courses and transitions into and out of MetS following continuous-time Markov chains. To form the cohort for our simulation we simulated 500,000 individual life courses. These individuals were split into four evenly sized groups (n = 125,000): females with low education; females with high education; males with low education; and males with high education. This number of life courses was sufficient to minimize the uncertainty associated with the Monte Carlo component of the model. We concluded that the error had reached an acceptable minimum, because the decrease in the standard deviation plateaued by the time the total sample size was 500,000 (Supplementary Figs. 1–3). Therefore, we determined that further increasing the sample size would likely not greatly reduce the uncertainty. The simulated life courses begin at the age of 18 years and end once the individuals reach the age of 65 years; mortality was not included in our microsimulation.

The simulated cohort is based on data from Lifelines, which is a multi-disciplinary, prospective, population-based cohort study examining, in a unique three-generation design, the health and health-related behaviours of 167,729 persons living in the North of the Netherlands. Lifelines employs a broad range of investigative procedures in assessing the biomedical, socio-demographic, behavioural, physical, and psychological factors which contribute to the health and disease of the general population, with a special focus on multi-morbidity and complex genetics. A detailed description of the study profile of Lifelines, the participant recruitment (between 2006 and 2013) and the data collection can be found elsewhere [21]. Briefly, Dutch speaking individuals aged 25–49 were asked to participate by their physicians. Those who accepted were subsequently asked to invite their family members. Individuals could also self-register through the Lifelines website. The first measurement wave took place between 2007 and 2014 and 2010–2014 in adults and children, respectively. During the first (T1) and fourth (T4) measurement wave, participants were asked to fill out questionnaires and, if aged 8 years or older, they also underwent physical exams.

The current study used data from 152,728 participants aged 18 years and older. In total, 59,479 individuals were excluded due to either being aged older than 65 years, being lost to follow-up, having missing data on MetS, or having missing values in more than 30% of the relevant variables. We excluded individuals older than 65 years because we did not account for mortality by MetS and educational level, as we have too little information to reliably estimate these rates and account for them in the microsimulation. However, as most deaths in the Dutch population occur above age 65 [23], not modeling mortality should not seriously compromise the validity of our results. This resulted in a final sample of 93,249 participants, of which 79,829 did not have MetS at baseline and 13,420 had MetS at baseline (Supplementary Fig. 4). We observed small differences between those included and excluded from the analysis. In particular, those who were included tended to be slightly younger, were more often female, were more highly educated, and had more favourable behaviours, compared to those excluded (Supplementary Table 1).

With the Lifelines data we then used multivariable logistic regression models to estimate three sets of rates from our selected sample: (1) MetS prevalence rates at age 18, (2) MetS incidence rates per 5-year age group between age 18 and age 65, and (3) MetS recovery rates per 5-year age group between age 18 and age 65. Prior to estimating the rates, missing values were imputed using the Multiple Imputation by Chained Equation (MICE) method (10 imputed samples drawn every 100 iterations) [24]. The imputation model included the independent variables, baseline age, sex, the modifiable variables, and the dependent variables. As the microsimulation models depend on continuous measures of age, these transition rates were then parameterized to account for continuous age; the internal validity of this procedure was assessed by checking the mean absolute error between our predicted and observed rates. The recovery rates were parameterized using a fourth-degree polynomial (mean absolute error: females with high education 0.0003; females with low education 0.0006; males with high education 0.0004; males with low education 0.0008), and the incidence rates were parameterized using a logit curve (mean absolute error: females with high education 0.0040; females with low education 0.0041; males with high education 0.0040; males with low education 0.0041). In our model we assume that these transition rates will persist into the future. Further details about the process used to handle missing data and estimate these rates are presented in Appendix A. The rates estimated using the Lifelines data were used in our microsimulation model to estimate the individual life courses of our synthetic cohort following a continuous-time, stochastic (random) process. We estimated from this synthetic cohort the life course prevalence, mean age of onset, and mean duration with MetS, separately by sex and educational level. *Life course prevalence* was derived by calculating the proportion of individuals who *ever* had MetS per sex- and educational levels. *Mean age of onset* was derived by calculating the average age at which individuals first develop MetS during the simulation; individuals who first entered the simulation with MetS and those who never developed MetS were excluded from the calculation of the mean age of onset. *Mean duration of MetS* was derived by calculating the average number of years spent with MetS amongst individuals who experienced MetS. Confidence intervals for these parameters were estimated by repeating each simulation 1000 times. Each run of the simulation included 50,000 synthetic individuals (n = 12,500 per sex and education group) and used unique transition rate curves. The curves were constructed by drawing new point estimates for each 5-year age group from a normal distribution, which were then parameterized. The normal distributions had a mean and standard deviation equal to the estimated transition rates and their standard errors. After estimating life course prevalence, mean age of onset, and mean duration with MetS in each run of the simulation we extracted the 2.5 and 97.5 percentiles to estimate the lower limit and upper limit of the 95% confidence interval, respectively.

### Assessing the impact of modifiable factors

To assess the impact of intervening on modifiable factors, we estimated counterfactual transition rates for the low education groups. These counterfactual transition rates allow us to consider what the educational inequalities in MetS would look like if the distribution of the modifiable factors in individuals with low education was the same as in individuals with high education. We used the user defined mimrgns command in Stata to estimate these counterfactual rates. The mimrgns command allows us to estimate the marginal effect, which is the change in the outcome due to a change in the modifiable factor(s) when the other variables are held constant [25]. The modifiable variables included in this analysis were occupational moderate-to-vigorous physical activity (MVPA), leisure time MVPA, smoking, alcohol intake, diet quality, sleep duration, network size, quality of social contacts, partner status, self-management skills, and health literacy. Both occupational and leisure time MVPA were included as literature has shown educational gradients in MetS exist for both of these forms of MVPA [26]. To identify which of these factors was most important, we estimated the mediating percentages of the modifiable variables. The four most important factors identified were smoking, alcohol intake, diet quality and health literacy. Details of the procedure used in the mediation analysis can be found in Appendix B.

Once identified, the most important modifiable factors were included in the logistic regression models used to estimate the age-specific counterfactual transition rates. We adjusted the distribution of the modifiable factors for individuals with low education to be the same as individuals with high education. This resulted in new transition rates for individuals with low education, which were then fed into the microsimulation models. We then followed the same procedure to estimate educational inequalities in MetS over the life course. In total, four counterfactual simulations were estimated (one per modifiable factor). We also estimated an additional simulation in which all modifiable factors were changed at once, which we refer to as the joint effect.

### Sensitivity analyses

In the sensitivity analyses, we assessed the role of very short-term transitioning into and out of MetS. If a transition was shorter than 6 months, the state was modified to reflect the first valid state lasting at least 6 months. This procedure discards any periods with MetS which may have occurred due to a temporary fluctuation above the cut-offs required to be classified as having MetS.

### Measures and procedures

Below, the operationalisation of the dependent variable and the main independent variable is described. The description of how all of the variables were measured in the Lifelines Cohort Study is presented in Supplementary Tables 2, and the operationalisation of the modifiable risk factors is presented in Supplementary Table 3.

#### Education

Educational level was coded into years of education, using the number of years it would take to complete each category by the fastest route possible [27]. In the microsimulations, low education is defined as junior general secondary education (10 years) and high education is defined as university education (16 years). Junior general secondary education is a rather minimal starting qualification on the labour market, which can be attained at the age of 16. These educational levels were selected because they allow for a comparison between two rather common but different educational levels.

#### Metabolic syndrome

MetS was present if at least three of the five components according to the National Cholesterol Education Program’s Adult Treatment Panel III (NCEP-ATPIII) were present [8]. The criteria are: (1) Waist circumference ≥ 102 cm in male or ≥ 88 cm in female; (2) Systolic blood pressure ≥ 130 mmHg or diastolic blood pressure ≥ 85 mmHg or use of blood pressure-lowering medication; (3) Triglycerides ≥ 150 mg/dL (1.7 mmol/l) or use of medication for elevated triglycerides; (4) High-density lipoprotein cholesterol < 40 mg/dL (1.0 mmol/L) in male or < 50 mg/dL (1.3 mmol/L) in female or use of lipid-lowering medication; (5) Fasting blood glucose level ≥ 100 mg/dL (≥ 5.6 mmol/l) or diagnosis of type 2 diabetes or use of blood glucose-lowering medication. The physical measurements and venous blood draws were conducted by trained research nurses using a standardized protocol [21]. Baseline medication use was classified according to the Anatomical Therapeutic Chemical coding scheme [28]; during follow-up it was classified according to the Lifelines questionnaire.

## Results

### Educational inequalities in MetS over the life course

**Fig. 1:**
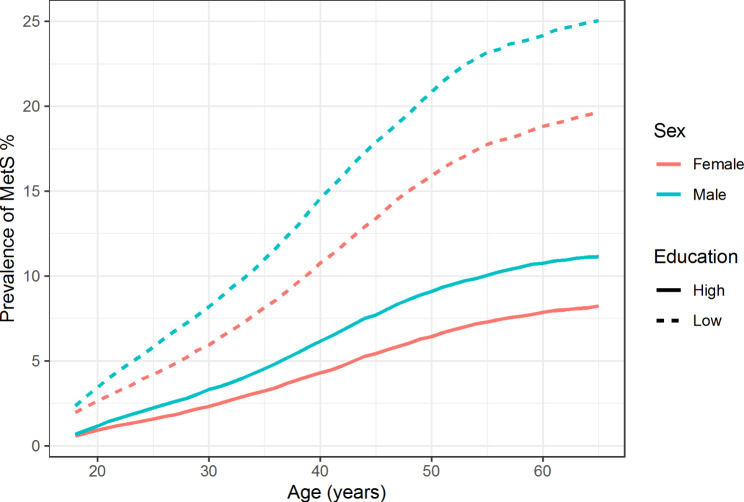
Age-specific prevalence of MetS stratified by sex and education *Note: The incidence rates for the simulation were estimated using 79,829 individuals (60.7% female), and the recovery rates were estimated using 13,420 individuals (48.0% female)

Based on the simulation, the life course prevalence of MetS is considerable among all distinguished groups (Table 1). However, compared to individuals with high education, individuals with low education are much more likely to ever experience MetS, develop MetS slightly earlier in life, and spend more time with MetS. Compared to individuals with high education, the life course prevalence of MetS is 26.8% points higher in females and 28.3% points higher in males with low education (Table 1 and Supplementary Table 11). Females and males with low education on average develop MetS 1.9 and 2.7 years earlier than their highly educated counterparts, respectively (Table 1 and Supplementary Table 11). Lastly, not only are individuals with low education more likely to ever experience MetS, but they also tend to spend more time living with MetS. On average females and males with low education spend an additional 2.3 and 2.9 years with MetS compared to their highly educated counterparts, respectively (Table 1 and Supplementary Table 11). Not only are the educational inequalities in males larger than in females across all measures, but on average males also have a higher life course prevalence (11.5% points), earlier mean age of onset (1 year), and longer mean duration of MetS than females (0.7 years). Lastly, while most low educated individuals experienced MetS, the prevalence at a given moment did not exceed 25.0% in individuals with low education and 11.1% in individuals with high education (Fig. 1). Overall, the results from the sensitivity analysis were in line with the results of the primary analysis (Supplementary Table 4).

**Table 1:** Educational inequalities in the development of MetS between ages 18 and 65 stratified by sex

Sex	Education	Simulation*	Life course prevalence of MetS: % (95%CI)	Mean age of onset of MetS: years (95%CI)	Mean duration of MetS: years (95%CI)
Females	High	Observed data	32.5 (30.7; 33.9)	45.4 (44.7; 46.2)	6.7 (6.4; 7.0)
	Low	Observed data	59.3 (57.8; 60.6)	43.5 (42.9; 44.1)	9.0 (8.7; 9.3)
		Counterfactual smoking	57.2 (55.6; 58.5)	43.7 (43.1; 44.3)	8.8 (8.6; 9.1)
		Counterfactual alcohol	58.0 (56.6; 59.4)	43.6 (43.0; 44.2)	8.8 (8.6; 9.1)
		Counterfactual health literacy	58.5 (57.0; 59.8)	43.6 (43.0; 44.2)	8.9 (8.7; 9.2)
		Counterfactual diet	58.7 (57.2; 60.0)	43.5 (43.0; 44.1)	8.9 (8.7; 9.2)
		Counterfactual joint effect	54.5 (53.0; 55.9)	43.9 (43.3; 44.5)	8.6 (8.3; 8.8)
Males	High	Observed data	43.2 (40.9; 44.5)	44.8 (44.0; 45.5)	7.0 (6.7; 7.2)
	Low	Observed data	71.5 (70.2; 72.9)	42.1 (41.6; 42.8)	9.9 (9.6; 10.2)
		Counterfactual smoking	69.4 (68.1; 71.0)	42.3 (41.8; 43.0)	9.7 (9.4; 9.9)
		Counterfactual alcohol	70.3 (69.0; 71.7)	42.2 (41.7; 42.9)	9.7 (9.4; 10.0)
		Counterfactual health literacy	70.7 (69.4; 72.2)	42.2 (41.6; 42.9)	9.8 (9.5; 10.1)
		Counterfactual diet	70.9 (69.6; 72.4)	42.1 (41.6; 42.8)	9.8 (9.5; 10.1)
		Counterfactual joint effect	66.9 (65.5; 68.5)	42.6 (42.1; 43.3)	9.3 (9.1; 9.6)

*Note: The results for the group with high education do not change in the counterfactual simulations, which is why they are not repeated in this table. The incidence rates for the simulation were estimated using 79,829 individuals (60.7% female), and the recovery rates were estimated using 13,420 individuals (48.0% female)

### The impact of intervening on modifiable factors

**Fig. 2:**
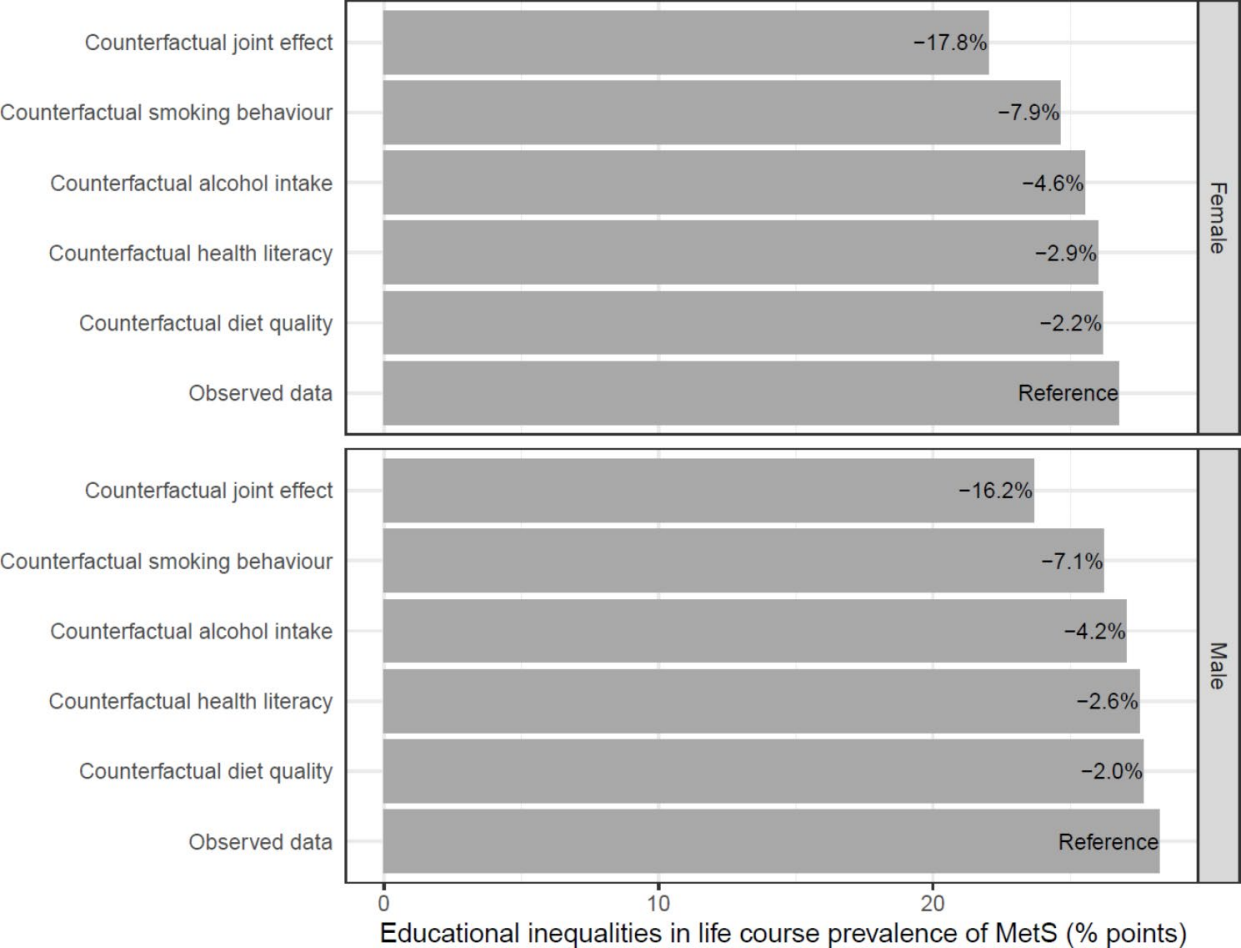
Potential impact of modifiable factors on educational inequalities in life course prevalence of MetS between ages 18 and 65 *Note: The bars show the additional proportion of individuals with low education who ever experience MetS compared to individuals with high education under different counterfactual conditions. The percentages shown on the bars represent the percentage reduction under the given counterfactual. The incidence rates for the simulation were estimated using 79,829 individuals (60.7% female), and the recovery rates were estimated using 13,420 individuals (48.0% female)

**Fig. 3:**
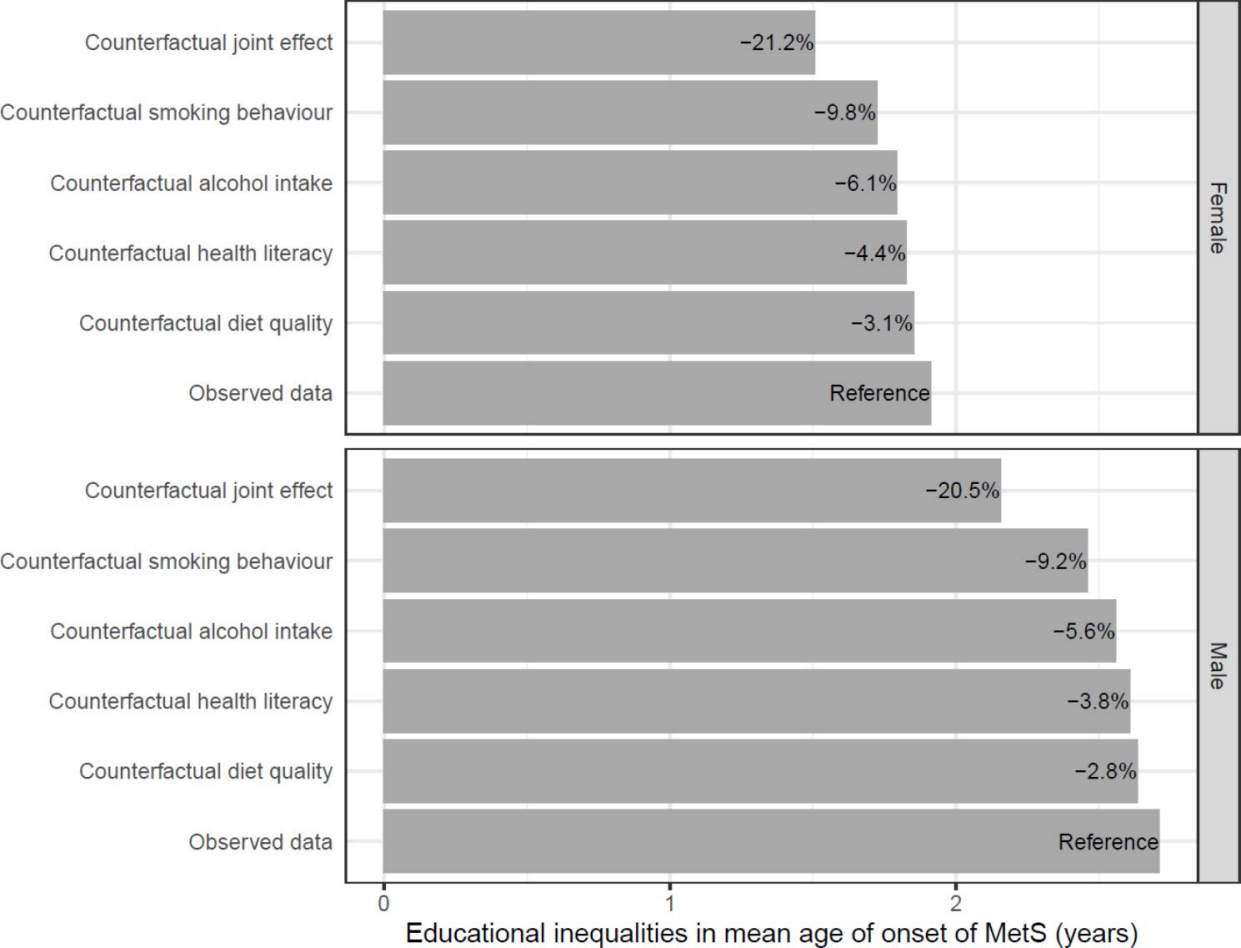
Potential impact of modifiable factors on educational inequalities in mean age of onset of MetS between ages 18 and 65 *Note: The bars show the difference in the mean age of onset of MetS between individuals with low and high education under different counterfactual conditions. Larger values indicate earlier age of onset amongst individuals with low education. The percentages shown on the bars represent the percentage reduction under the given counterfactual. The incidence rates for the simulation were estimated using 79,829 individuals (60.7% female), and the recovery rates were estimated using 13,420 individuals (48.0% female)

**Fig. 4:**
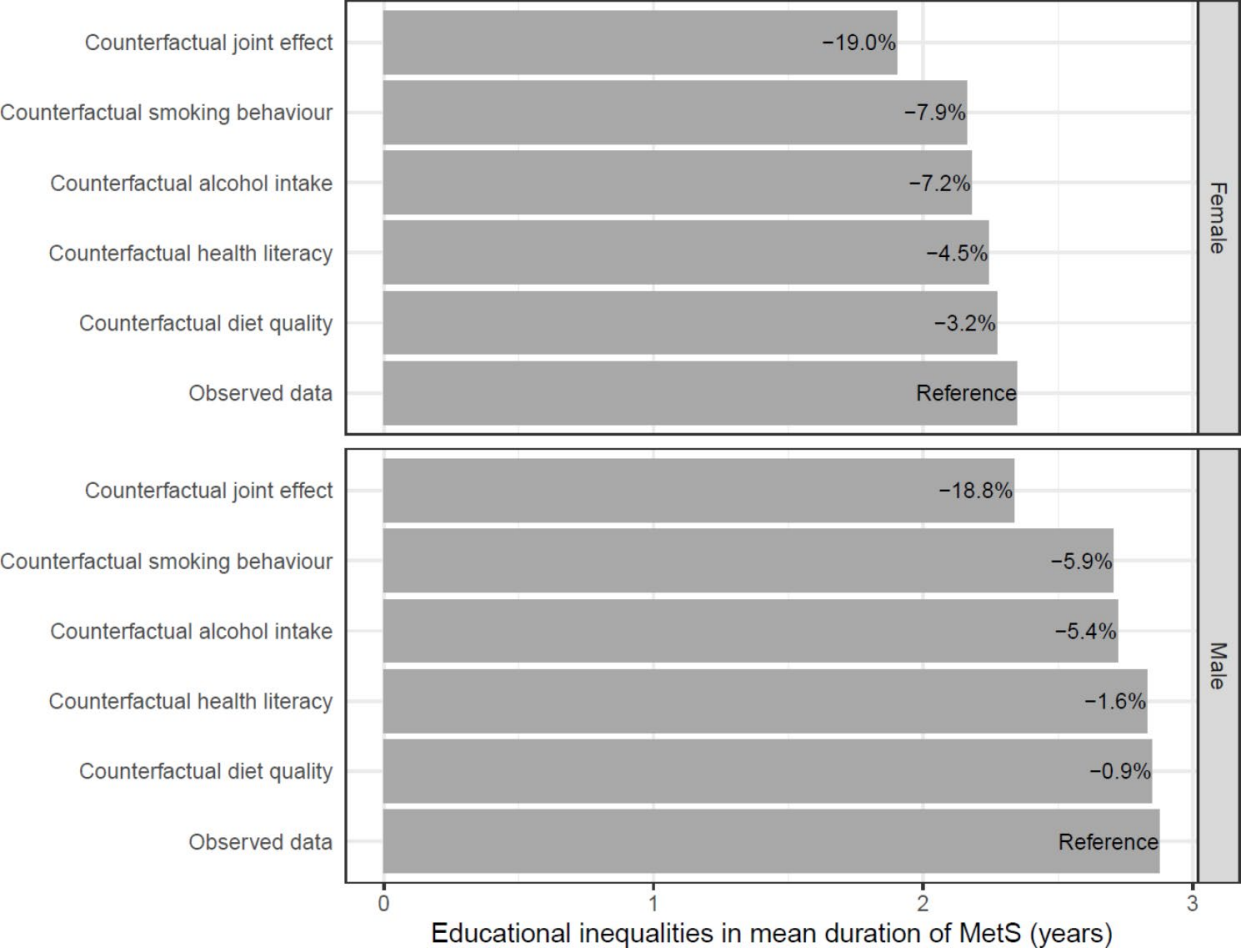
Potential impact of modifiable factors on educational inequalities in mean duration of MetS between ages 18 and 65 *Note: The bars show the additional number of years spent with MetS for individuals with low education compared to individuals with high education under different counterfactual conditions. The percentages shown on the bars represent the percentage reduction under the given counterfactual. The incidence rates for the simulation were estimated using 79,829 individuals (60.7% female), and the recovery rates were estimated using 13,420 individuals (48.0% female)

The mediation analyses identified that smoking, alcohol intake, health literacy, and diet quality were the most important modifiable factors to reduce educational inequalities in MetS (Supplementary Tables 5–10). In general, individuals with high education had a more favorable distribution of the modifiable factors compared to individuals with low education (Table 2). These mediators were then used in the counterfactual simulations, which demonstrate that changes in the modifiable factors reduce the educational inequalities in the life course prevalence, timing, and duration of MetS (Figs. 2, 3 and 4 and Supplementary Table 11). These changes refer to changing the distribution of the modifiable factors amongst individuals with low education to match that seen amongst individuals with high education. For example, this would entail a reduction in the amount of smoking in the group with low education (Table 2).

**Table 2:** Distribution of modifiable factors by educational level (n = 93,249; 58.9% Female)

Modifiable factors	Low education (%*)	High education (%*)
Smoking		
Never	37.5	63.4
Former	38.9	24.8
Current	23.7	11.8
Health literacy		
Low	32.4	8.5
High	67.6	91.5
Alcohol Consumption		
None	23.3	11.8
Moderate	40.9	59.6
Problematic	35.8	40.4
Diet		
Healthy	8.5	13.0
Moderate	80.3	81.6
Unhealthy	11.3	5.4

*Note due to rounding percentages may not sum to 100

Amongst the independent modifiable factors, smoking had the largest effect on educational inequalities in MetS. If smoking behaviours in females with low education were changed to reflect the patterns seen in females with high education, the life course prevalence would decrease from 59.3 to 57.2%, which is an 7.9% reduction in the educational inequalities (Table 1; Fig. 2 and Supplementary Table 11). For males, the educational inequalities in the life course prevalence of MetS would decrease by 7.1%. Changes to smoking would increase the mean age of onset in females with low education from 43.5 years to 43.7 years, which is an 9.8% reduction in the educational inequalities (Table 1; Fig. 3). For males, the mean age of onset would increase from 42.1 years to 42.3 years, which is a 9.2% decrease in the educational inequalities in the mean age of onset. Similarly, changes to smoking would decrease the mean duration of MetS in females with low education from 9.0 to 8.8 years, which is a 7.9% reduction in the educational inequalities (Table 1; Fig. 4). For males, the mean duration of MetS would decrease from 9.9 years to 9.7 years, which is a 5.9% decrease. Similarly, changes to alcohol intake, health literacy and diet quality also reduced educational inequalities in MetS, but to a lesser extent than changes in smoking behaviour (Figs. 2, 3 and 4 and Supplementary Table 11).

The largest reduction in educational inequalities was seen when all the modifiable factors in individuals with low education were changed to reflect the patterns seen in individuals with high education; the inequalities in the life course prevalence, mean age of onset, and mean duration of MetS would be reduced by 17.8%, 21.2%, and 19.0% in females and 16.2%, 20.5%, and 18.8% in males, respectively. Again, the results from the sensitivity analysis (Supplementary Figs. 5–7 and Supplementary Table 12) showed similar results as the primary analysis.

## Discussion

We aimed to simulate educational inequalities in the development of MetS across the life course, and to assess the impact of intervening on modifiable factors that contribute to these inequalities. We found that individuals with low education were much more likely to ever experience MetS, develop MetS somewhat earlier in life, and spent more time with MetS than individuals with high education; these educational inequalities were generally larger between males with low and high education than between females with low and high education. Interventions targeting the modifiable factors included in this study, especially smoking, could help reduce these educational inequalities.

If current age-specific transition rates persist into the future, we estimate that the prevalence of MetS is going to increase dramatically as the population ages, especially amongst males and individuals with lower education. Moreover, the worldwide increasing prevalences of overweight and obesity will contribute to this dramatic increase [9]. Our estimates suggest that 71.5% of males with low education will develop MetS at some point between the ages of 18 and 65, whereas 43.2% of males with high education will develop MetS. For females with low and high education, the risks are lower, but still substantial, 59.3% and 32.5%, respectively. These findings are consistent with studies on the cross-sectional prevalence and incidence of MetS by educational level [2, 5]. Our results also show that females and males with low education develop MetS 1.9 and 2.7 years earlier than their counterparts with high education, respectively. This is consistent with Dutch healthy life expectancies of the general population; higher educated individuals live longer in good health [23]. Further, our estimates suggest that females and males with low education spend an additional 2.3 and 2.9 years with MetS compared to their highly educated counterparts, respectively. These estimates are supported by previous longitudinal studies suggesting that individuals with low education had a greater risk to develop MetS and were less likely to recover from MetS, compared to individuals with high education [2, 5, 10]. According to our results, individuals with low education have a triple burden for MetS as they are more likely to develop MetS, develop MetS earlier in life, and spend more time with MetS than individuals with high education. We found that about 20% of the educational inequalities in MetS prevalence, timing and duration are related to differences in smoking, alcohol intake, diet quality, and health literacy levels. Despite the fact that our model is a simplification of reality, as all modifiable factors are interrelated (e.g., health literacy could influence lifestyle [29]), we do see interesting opportunities for intervening on the individual modifiable factors.

An important strength is the use of microsimulation models to assess educational inequalities in the development of MetS across the life course and the role of modifiable factors. Most traditional epidemiological studies considered educational inequalities in MetS development and recovery by examining the proportion in the population at one point in time. As seen in our results, the proportion of individuals with MetS in the population at one point in time does not reflect the proportion who will ever experience MetS during their life course; the educational inequalities in life course prevalence were larger than the inequalities at a given point in time. Educational inequalities in the prevalence of MetS over the life course do not only depend on differences in the incidence, but also on differences in recovery from MetS. Regarding data, we used data from Lifelines, a large population based cohort [21, 30], to estimate the parameters for our simulation.

Clearly, our microsimulation approach is based on a number of assumptions, each of which should be scrutinised. One important assumption underlying the microsimulation model is that current transition rates will persist into the future, which may not be the case. Second, we did not account for mortality. Individuals with MetS have a higher risk of dying prematurely [7]; therefore, the duration of MetS would be lower if we included mortality, which would have resulted in smaller educational inequalities in the duration of MetS. This does not imply that ignoring mortality results in overestimating educational inequalities, though. In contrast, the smaller educational inequalities in a model including mortality would not be caused by more years without MetS but by more life years lost. To keep our microsimulation as realistic as possible we decided to limit our analysis to those aged 18 to 65, as in the Netherlands most individuals survive to age 65 [23]. Third, we compared just two educational levels, i.e. those with a rather minimal starting qualification on the labour market (i.e., having a diploma that could be acquired at the age of 16), with those having a university education. If we would have included intermediate levels of education in the analysis, differences between specific educational groups would have been smaller. Nonetheless, our analysis includes two educational levels that are quite common in the Netherlands. Fourth, our data are from the Netherlands, a country with a rather strong welfare state and highly subsidized health care system. Finding large educational inequalities in this setting suggests that these inequalities might be larger in countries that are either less highly developed, less egalitarian or both.

Regarding potential limitations, it is important to note that there were differences between those included and excluded from the analysis. The sample used to estimate the transition rates tended to be slightly younger, contained a greater proportion of females, was more highly educated, and had more favourable modifiable factors. Due to this, we may have a somewhat lower representation of those with lower levels of educational attainment and more unfavourable values of the modifiable factors. If anything, this may have resulted in some underestimation of the educational inequalities in MetS.

Reducing educational inequalities in the prevalence, timing and duration of MetS should be a main focus of interventions and treatment. One approach would be to focus specifically on improving educational attainment in vulnerable groups. In the Netherlands, young adults who leave the educational system without a diploma would be considered a particularly vulnerable group. Existing policies have reduced the proportion of individuals aged 18 to 25 years who leave the educational system without a diploma from 9.0% to 2013 to 5.6% in 2022 [31]. Additional gains could be made by targeting this group. An alternative approach could be to implement more general policies to educate young people about healthy lifestyles, which could benefit individuals in the lower echelons of the educational system. For example, policies, tailor-made interventions, and treatment for individuals with low education could focus on reducing smoking and alcohol intake and increasing health literacy and diet quality in the short run (e.g., via sugar or smoking taxes). These factors were all shown to partially reduce educational inequalities in the development of MetS over the life course. Amongst these factors smoking behaviour was by far the most important and should be prioritized in policy and treatment. Additionally, further research is needed to explore what other factors account for these educational inequalities, of which work and income are two candidates.

## Conclusion

This study fills an important gap in our understanding of how inequalities in MetS develop over time and has policy implications for targeting of prevention efforts. Individuals with low education are more likely to ever experience MetS, develop MetS earlier in life, and spend more time with MetS compared to individuals with high education. These inequalities could partially be reduced by targeting smoking, and to a lesser extent alcohol consumption, health literacy and diet quality.

### Electronic supplementary material

Below is the link to the electronic supplementary material.

Supplementary Material 1: Supplementary Data

Supplementary Material 2: STROBE Checklist

## Data Availability

The generated dataset is not publicly available as it is created and used under license from the Lifelines Cohort Study. Data from the Lifelines Cohort Study is available on request (www.lifelines.nl). However, the transition rates which were estimated using the Lifelines dataset and serve as the input for the microsimulation models are available online. The code for the estimation of the prevalence rates, transition rates, and microsimulation models is available online (https://github.com/a-lepe/TRANSSES_mets_simulation).

## Abbreviations

CVD: Cardiovascular disease
MetS: Metabolic syndrome
MICE: Multiple Imputation by Chained Equation
MVPA: Moderate-to-Vigorous Physical Activity
NCEP-ATPIII: Cholesterol Education Program’s Adult Treatment Panel III
