# Supplementary material for: Educational inequalities in metabolic syndrome prevalence, timing, and duration amongst adults over the life course: a microsimulation analysis based on the lifelines cohort study
Source: Int J Behav Nutr Phys Act. 2023 Sep 4;20:104. doi: 10.1186/s12966-023-01495-1 (PMC10478481; doi:10.1186/s12966-023-01495-1)
Supplement: Supplementary file 1 — Supplementary Material 1: Supplementary Data [file 12966_2023_1495_MOESM1_ESM.docx]

**SUPPLEMENTARY DATA**

1. **Appendix A. Description of the process used to estimate and parameterize the rates for the microsimulations.**
2. **Appendix B. Description of the process used to estimate the mediating percentages used to derive the most important modifiable factors.**
3. **Supplementary Tables.**

Table S1. Descriptive characteristics of the study population with and without MetS at baseline (n = 93,249).

Table S2. Measurement in the Lifelines Cohort Study of the variables used in the analyses.

Table S3. Operationalisation of the modifiable variables included in the mediation analyses.

Table S4. Educational inequalities in the development of MetS between ages 18 and 65 stratified by sex. Sensitivity analysis in which transitions with a duration of less than six months were discarded.

Table S5. Multivariable logistic regression analysis of the direct associations between education, lifestyle, social, and psychological factors and incident metabolic syndrome (n = 79,829).

Table S6. Multivariable mediation analysis of lifestyle, social, and psychological factors in the associations between education and incident metabolic syndrome using the Karlson-Holm-Breen method (n = 79,829).

Table S7. Multivariable logistic regression analysis of the direct associations between education, lifestyle, social, and psychological factors and having recovered from metabolic syndrome (n = 13,420).

Table S8. Multivariable mediation analysis of lifestyle, social, and psychological factors in the associations between education and having recovered from metabolic syndrome using the Karlson-Holm-Breen method (n = 13,420).

Table S9. Multivariable logistic regression analysis of the direct associations between education and the most important mediating factors and incident and having recovered from metabolic syndrome (n = 93,249).

Table S10. Multivariable mediation analysis of the most important mediating factors in the association between education and incident and having recovered from metabolic syndrome using the Karlson-Holm-Breen method (n = 93,249).

Table S11. Educational differences in the development of MetS over the life course stratified by sex.

Table S12. Educational differences in the development of MetS over the life course stratified by sex: sensitivity analysis.

1. **Supplementary Figures.**

Figure S1. Monte Carlo variance associated with the estimate of the life course prevalence of MetS.

Figure S2. Monte Carlo variance associated with the estimate of the mean age of onset of MetS.

Figure S3. Monte Carlo variance associated with the estimate of the mean duration of MetS.

Figure S4. Flowchart of the population with and without MetS at baseline.

Figure S5. Potential impact of modifiable factors on educational inequalities in life course prevalence of MetS between ages 18 and 65. Sensitivity analysis in which transitions with a duration of less than six months were discarded. Results presented separately for each simulation.

Figure S6. Potential impact of modifiable factors on educational inequalities in mean age of onset of MetS between ages 18 and 65. Sensitivity analysis in which transitions with a duration of less than six months were discarded. Results presented separately for each simulation.

Figure S7. Potential impact of modifiable factors on educational inequalities in mean duration of MetS between ages 18 and 65. Sensitivity analysis in which transitions with a duration of less than six months were discarded. Results presented separately for each simulation.

Figure S8. Observed (points) and expected (lines) values of transition rates for males and females by education level.

1. **Appendix A. Description of the process used to handle missing data and estimate the rates for the microsimulations.**

A description of our sample from the Lifelines Cohort Study including the amount of missing data can be found in Supplementary Table 1. Missing values were imputed using the Multiple Imputation by Chained Equation (MICE) method (10 imputed samples drawn every 100 iterations) ^20^**.** Participants were excluded if three or more MetS indicators were missing or based on three or four indicators present it was not possible to determine with certainty whether or not they had MetS. The imputation model included the independent variables, baseline age, sex, the modifiable variables, and the dependent variables.

This data were used to estimate the sex- and education specific prevalence of MetS at the age of 18 years. This prevalence was calculated by estimating the probability of MetS at baseline in the group of participants ≤27 years for participants aged 18 years, per sex (male or female) and per education group (10 or 14 years) using logistic regression models. The model takes the form:

$$log\left( \frac{P(MetS)}{1-P(MetS} \right)=a+b1*Age+b2*Sex+b3*Education$$

We also used the Lifelines data to estimate the age, sex, and education specific incidence and recovery rates of MetS using 5-year age groups. The incidence and recovery rates were estimated using logistic regression models which included age-, sex-, education, smoking status, health literacy, alcohol use, and diet quality as predictors of MetS status during the second assessment.

As the microsimulation models depend on continuous measures of age these transition rates were then parameterized to account for continuous age. The type of parametric model for each transition rate was chosen based on the goodness of fit of the model with the observed data. Goodness-of-fit was assessed by visual inspection of the graph of the model and observed data (Supplementary Figure 8). The best fitting models were a fourth-degree polynomial for recovery rates (mean absolute error: females with high education 0.0003; females with low education 0.0006; males with high education 0.0004; males with low education 0.0008), and a logit curve for the incidence rates (mean absolute error: females with high education 0.0040; females with low education 0.0041; males with high education 0.0040; males with low education 0.0041).

**2. Appendix B. Description of the process used to estimate the mediating percentages used to derive the most important modifiable factors.**

*Population selection to estimate the mediating percentages*

The current study used data from 152,728 participants aged 18 years and older. We applied the same exclusion criteria as used for the microsimulation: participants aged older than 65 were excluded (n=10,921). Participants with missing data on MetS at baseline (n=4,907) or follow-up (n=41,871), participants who were lost to follow-up (n=17) or who had >30% missing values on the relevant variables in this study (n=2,420) were excluded from the analyses. This resulted in a final sample of 92,592 participants, of which 79,272 did not have MetS at baseline and 13,320 had MetS at baseline.

*Statistical analysis*

Missing values were imputed using the Multiple Imputation by Chained Equation (MICE) method (10 imputed samples drawn every 100 iterations) ^20^**.** Participants were excluded if three or more MetS indicators were missing or based on three or four indicators present it was not possible to determine with certainty whether or not they had MetS. The imputation model included the independent variables, baseline age, sex, the modifiable variables, and the dependent variables. With the imputed datasets we then used a multivariable logistic regression analysis, controlling for age, sex, and time between baseline and the second assessment, to estimate the direct associations between education, the modifiable factors and both incident MetS and going into remission. The total, direct and indirect associations between education and both incident MetS and going into remission via the modifiable factors and the mediating percentages of the modifiable factors were estimated using the Karlson-Holm-Breen (KHB) method ^21^. The imputation of missing data and the mediation analyses were performed using StataMP 13 (64-bit). P-values<0.05 were considered to be statistically significant.

**3. Supplementary Tables.**

**Table S1.** Descriptive characteristics of the study population as part of the Lifelines cohort with and without MetS at baseline (n = 93,249).

| **Characteristics** | **Lifelines population (n=152,728)^a^** | **Excluded population (n=59,479)^a^** | **Study population (n = 93,249)^a^** | **Population without MetS at baseline (n = 79,829)^a^** | **Population with MetS at baseline (n = 13,420)^a^** |
| --- | --- | --- | --- | --- | --- |
| Age (years), mean (SD) | 44.6 (13.1) | 45.6 (15.8) | 44.0 (11.1) | 43.1 (11.0) | 49.6 (9.7) |
| Missing | 0 | 0 | 0 | 0 | 0 |
| Sex (female) | 58.5 | 57.9 | 58.9 | 60.7 | 48.0 |
| Missing | 0 | 0 | 0 | 0 | 0 |
| Education (years of education) |  |  |  |  |  |
| Primary school not finished (5) | 0.6 | 0.9 | 0.4 | 0.3 | 1.0 |
| Primary education (6) | 2.3 | 3.9 | 1.3 | 1.1 | 2.4 |
| Lower or preparatory secondary vocational education (9) | 13.3 | 14.9 | 13.3 | 10.9 | 20.3 |
| Junior general secondary education (10) | 13.6 | 14.7 | 12.9 | 12.3 | 16.4 |
| Secondary vocational education or senior general secondary education (12) | 38.5 | 36.0 | 40.2 | 40.7 | 36.6 |
| Higher vocational education (15) | 23.2 | 20.4 | 24.9 | 26.1 | 17.8 |
| University education (16) | 6.0 | 5.4 | 6.3 | 6.8 | 3.4 |
| Missing | 2.6 | 3.7 | 1.8 | 1.8 | 2.2 |
|  |  |  |  |  |  |
| **Modifiable factors** |  |  |  |  |  |
| Smoking |  |  |  |  |  |
| Never smoker | 43.1 | 39.3 | 45.5 | 47.3 | 35.0 |
| Past smoker | 29.7 | 27.4 | 31.2 | 29.9 | 39.0 |
| Current smoker | 20.5 | 21.7 | 19.8 | 19.3 | 22.6 |
| Missing | 6.7 | 11.7 | 3.5 | 3.5 | 3.5 |
| Alcohol intake^b^ |  |  |  |  |  |
| No alcohol intake | 19.2 | 18.3 | 19.7 | 19.0 | 24.1 |
| Moderate alcohol intake | 40.7 | 36.3 | 43.5 | 44.6 | 36.7 |
| Excessive alcohol intake | 34.6 | 31.1 | 36.7 | 36.3 | 39.1 |
| Missing | 5.6 | 14.3 | 0.1 | 0.1 | 0.1 |
| Diet quality^c^ |  |  |  |  |  |
| Poor | 7.2 | 6.6 | 7.6 | 7.7 | 6.8 |
| Moderate | 68.2 | 62.0 | 72.2 | 72.2 | 72.2 |
| High | 9.3 | 9.7 | 9.0 | 9.0 | 9.4 |
| Missing | 15.3 | 21.8 | 11.2 | 11.1 | 11.7 |
| Health literacy at T3^d^ |  |  |  |  |  |
| Low | 17.0 | 11.6 | 20.5 | 19.5 | 26.0 |
| High | 44.9 | 26.8 | 56.4 | 57.2 | 52.1 |
| Missing | 38.1 | 61.6 | 23.1 | 23.3 | 21.9 |

MetS: metabolic syndrome; SD: standard deviation; LLDS: Lifelines Diet Score; ^a^ % are presented, unless indicated otherwise; ^b^ According to the guidelines of the Dutch Health Council; ^c^ According to the Lifelines Diet Score (‘poor’ = 1-16, ‘moderate’ = 17-32, ‘high’ = 33-48); ^d^ Measured with the Brief Health Literacy Screening (BHLS) on a continuous scale (3-15) which was categorized in low health literacy 3-12 and high health literacy 13-15.

**Table S2.** Measurement in the Lifelines Cohort Study of the variables used in the analyses.

| **Variables** | **Measured in the Lifelines Cohort Study** |
| --- | --- |
| Education | ‘What is the highest level of education you have completed?’ Participants had eight answer options, including ‘No education’, ‘Primary education’, ‘Lower or preparatory secondary vocational education’, ‘Junior general secondary education’, ‘Secondary vocational education or work-based learning pathway’, ‘Senior general secondary education, pre-university secondary education’, ‘Higher vocational education’ and ‘University education’. |
| Waist circumference | During the physical examination using the SECA 200 measuring tape. |
| Blood pressure | Measurements at baseline consisted of 10 measurements during 10 minutes using the Dinamap PRO 100V2. During the second assessment measurements consisted of three measurements using the Dinamap PRO 100V2. At both occasions, blood pressure was calculated as the average of the last two measurements. |
| Triglyceride | Measured during a laboratory assessment on the day of the fasten blood sample collection. |
| High-density lipoprotein cholesterol | Measured during a laboratory assessment on the day of the fasten blood sample collection. |
| Fasting blood glucose | Measured during a laboratory assessment on the day of the fasten blood sample collection. If participants indicated they did not fast before the blood sample collection, their blood glucose values were assumed not to be valid if they exceeded 5.6 mmol/L according to the cut-off for having MetS and were interpreted as missing value. The determination of type 2 diabetes was based on self-reported questionnaires. |
| Medication use at baseline | The participant was asked about medication use through a questionnaire and by bringing used medications to the research site at the time of the physical examination. All prescribed medications were classified according to the Anatomical Therapeutic Chemical (ATC) coding scheme ^14^**.** |
| Medication use at the second assessment | ‘Do you use prescribed medication?’, participants answering yes or no. Specific information about the prescribed medication was not available at the second assessment. Measurement of these components in the Lifelines Cohort Study was performed in the same way at baseline and the second assessment unless indicated otherwise. |
| Occupational moderate-to-vigorous physical activity | Measured using the Short QUestionnaire to Assess Health enhancing physical activity (SQUASH) ^22^ with questions about occupational, cycling, gardening and sports activities. Complying to at least 150 minutes per week moderate-to-vigorous activity at work ^23^. |
| Leisure time moderate-to-vigorous physical activity | Measured using the Short QUestionnaire to Assess Health enhancing physical activity (SQUASH) ^22^ with questions about occupational, cycling, gardening and sports activities. Complying to at least 150 minutes per week moderate-to-vigorous activity during leisure time ^23^. |
| Smoking | ‘Have you ever smoked for a full year?’ and ‘Do you smoke now, or have you smoked in the past month?’. |
| Alcohol | Measured using the Food Frequency Questionnaire (FFQ) ^24^ with the questions ‘How often did you drink alcoholic drinks in the past month?’ and ‘How many glasses (i.e. alcoholic drinks) did you drink per day on average?’. |
| Diet | The Lifelines Diet Score (LLDS) is based on the 2015 Dutch Dietary Guidelines ^25^ and measures the consumption of nine food groups (vegetables, fruit, whole grain products, legumes, nuts, fish, oils- and soft margarines, unsweetened dairy, coffee and tea) with positive and three food groups (red- and processed meat, butter- and hard margarines and sugar-sweetened beverages) with negative health effects. |
| Sleep | ‘On average how many hours do you sleep per full day (24 hours)?’ |
| Network size | ‘With on average how many different people do you have contact over a period of two weeks?’ |
| Quality of social contacts | Examples of the items assessed are ‘Do you feel that people really love you?’, ‘Do you feel useful to others?’ and ‘Are you known for the things you have accomplished?’. |
| Partner status | ‘Do you have a partner?’ |
| Self-management skills | The skills are:   1. Taking the initiative (active-motivational ability) 2. Self-efficacy beliefs (cognitive ability) 3. Investment behaviour (active-motivational ability) 4. A positive frame of mind (cognitive ability) 5. Multifunctionality or resources (resource-combining ability) 6. Variety in resources (resource-combining ability) |
| Health literacy | Three questions:  1. ‘How often do you have trouble understanding you medical situation because you have difficulty with the written information?’  2. ‘How sure are you of yourself when you fill out medical forms?’  3. ‘How often does someone help you with reading information materials from the hospital or another healthcare provider?’ Reversing the scores on the first and third question and adding up the scores on all questions led to a continuous scale (3–15). |

**Table S3.** Operationalisation of the modifiable variables included in the mediation analyses.

| **Variable** | **Operationalisation in this study** |
| --- | --- |
| Physical activity | These factors were defined by occupational moderate-to-vigorous physical activity (MVPA), leisure time MVPA, smoking, alcohol intake, diet quality and sleep duration at baseline. Occupational MVPA and leisure time MVPA were measured using the SQUASH questionnaire and dichotomized based on whether participants completed at least 150 minutes MVPA per week respectively at work or during leisure time ^22,23^. |
| Smoking | Smoking habits were categorized as never, former or current smoker. |
| Alcohol intake | Alcohol intake measured with the Food Frequency Questionnaire was categorized as no alcohol intake, moderate alcohol intake defined as one glass of alcohol or less per day on average, without binge drinking (more than three glasses alcohol on one day for females and more than four glasses alcohol on one day for males) or excessive alcohol intake defined as more than one glass of alcohol per day on average or binge drinking ^24,26^. |
| Diet quality | Diet quality was based on the Lifelines Diet Score (LLDS) which was based on the 2015 Dutch Dietary Guidelines ^25,27^. The LLDS is calculated as the sum of positive and negative food group quintile scores (range 0-48) and relative to the diet quality of the Lifelines population. Higher scores indicate a healthier diet. In the current study, there were no participants with a LLDS of 0 and therefore participants were divided into three groups, according to their LLDS, ‘poor’ diet quality (LLDS 1-16), ‘moderate’ diet quality (LLDS 17-32) or ‘high’ diet quality (LLDS 33-48). |
| Sleep duration | Sleep duration per full day was categorized as long (>9 hours), normal (7-9 hours) or short (<7 hours). |
| Network size | Network size was categorized as <5, 5-9, 10-14, ≥15 contacts |
| Quality of social contacts | The quality of social contacts was assessed using nine items from the Social Production Function Instrument for Level of Well-Being (SPF-IL) measuring ‘affection’, ‘behavioural confirmation’ and ´status´ ^28^. Answers could be given on a four-point scale ranging from never (0) to always (3). A sum score was calculated (range 0–27) with higher scores indicating higher social need fulfilment. Social need fulfilment was categorized as ‘low’(< =14 points),‘middle’(15–17 points) and ‘high’(18–27 points). |
| Partner status | Partner status was categorized as having a partner or not during the baseline assessment. |
| Self-management skills | Self-management skills were measured with the Self-Management Ability Score (SMAS) ^29,30^. The SMAS measures six self-management skills, resulting in subscores per skill as well as a total sum score. The six skills are based on the theory of the self-management of well-being ^31^. The SMAS sum score was categorized into ≤66 and >67. The categorization for self-management was based on the capturing of the non-linear relationship with education and resulted in a sufficient number of individuals in each category. |
| Health literacy | Health literacy was measured with the Brief Health Literacy Screening (BHLS) ^32^, indicating the health literacy with three questions on understanding medical information. Participants answered these questions on a 5-point scale and this led to a continuous scale (3-15) which was categorized in low health literacy 3-12 and high health literacy 13-15. The categorization for health literacy was based on the capturing of the non-linear relationship with education and resulted in a sufficient number of individuals in each category. |

**Table S4.** Educational inequalities in the development of MetS between ages 18 and 65 stratified by sex. Sensitivity analysis in which transitions with a duration of less than six months were discarded.

| **Sex** | **Education** | **Simulation*** | **Life course prevalence of MetS: %**  **(95%CI)** | **Mean age of onset of MetS: years (95%CI)** | **Mean duration of MetS: years (95%CI)** |
| --- | --- | --- | --- | --- | --- |
| Females | High | Observed data | 30.4 (28.6; 31.6) | 45.4 (44.5; 46.0) | 7.1 (6.9; 7.4) |
|  | Low | Observed data | 56.7 (55.2; 58.1) | 43.6 (42.8; 44.0) | 9.4 (9.1; 9.7) |
|  | Low | Counterfactual smoking | 54.6 (53.0; 56.0) | 43.8 (43.0; 44.1) | 9.2 (9.0; 9.5) |
|  | Low | Counterfactual alcohol | 55.5 (53.9; 56.8) | 43.7 (42.9; 44.1) | 9.2 (9.0; 9.5) |
|  | Low | Counterfactual health literacy | 55.9 (54.3; 57.3) | 43.7 (42.8; 44.0) | 9.3 (9.1; 9.6) |
|  | Low | Counterfactual diet | 56.2 (54.6; 57.4) | 43.7 (42.8; 44.0) | 9.3 (9.1; 9.6) |
|  | Low | Counterfactual joint effect | 52.0 (50.4; 53.4) | 44.0 (43.2; 44.4) | 9.0 (8.7; 9.2) |
| Males | High | Observed data | 40.6 (38.4; 41.8) | 44.9 (43.9; 45.2) | 7.4 (7.2; 7.7) |
|  | Low | Observed data | 68.9 (67.5; 70.4) | 42.3 (41.4; 42.7) | 10.2 (10.0; 10.5) |
|  | Low | Counterfactual smoking | 66.9 (65.4; 68.3) | 42.6 (41.7; 42.9) | 10.1 (9.8; 10.3) |
|  | Low | Counterfactual alcohol | 67.7 (66.2; 69.1) | 42.5 (41.6; 42.8) | 10.1 (9.8; 10.4) |
|  | Low | Counterfactual health literacy | 68.1 (66.7; 69.6) | 42.4 (41.5; 42.7) | 10.2 (9.9; 10.4) |
|  | Low | Counterfactual diet | 68.3 (66.9; 69.8) | 42.4 (41.5; 42.7) | 10.2 (9.9; 10.4) |
|  | Low | Counterfactual joint effect | 64.2 (62.8; 65.8) | 42.8 (42.0; 43.2) | 9.7 (9.4; 10.0) |

*Note: The results for the group with high education do not change in the counterfactual simulations, which is why they are not repeated in this table. The incidence rates for the simulation were estimated using 79,829 individuals (60.7% Female), and the recovery rates were estimated using 13,420 individuals (48.0% Female)

**Table S5.** Multivariable logistic regression analysis of the direct associations between education, lifestyle, social, and psychological factors and incident MetS (n = 79,829).

|  | **OR (95% CI)** |
| --- | --- |
| **Path 1. Education and MetS** | 0.90 (0.89, 0.91)* |
| **Path 2. Education and lifestyle, social, and psychological factors** |  |
| **Lifestyle factors** |  |
| Occupational moderate-to-vigorous physical activity |  |
| No | 1.26 (1.24, 1.27)* |
| Leisure time moderate-to-vigorous physical activity |  |
| No | 0.96 (0.95, 0.96)* |
| Smoking |  |
| Former | 0.93 (0.92, 0.94)* |
| Current | 0.84(0.84, 0.85)* |
| Alcohol intake |  |
| No | 0.88 (0.88, 0.89)* |
| Excessive | 0.96 (0.95, 0.97)* |
| Diet quality |  |
| Moderate | 0.86 (0.85, 0.87)* |
| Poor | 0.73 (0.72, 0.74)* |
| Sleep duration |  |
| <7 | 0.98 (0.97, 0.99)* |
| >9 | 0.85 (0.83, 0.88)* |
| **Social factors** |  |
| Network size |  |
| <5 | 0.87 (0.86, 0.89)* |
| 5-9 | 0.97 (0.96, 0.98)* |
| >=15 | 1.01 (1.00, 1.01) |
| Quality of social contacts |  |
| <=14 | 0.87 (0.86, 0.88)* |
| 15-17 | 0.97 (0.96, 0.98)* |
| Partner status |  |
| No | 0.95 (0.94, 0.96)* |
| **Psychological factors** |  |
| Self-management skills |  |
| <=66 | 0.90 (0.89, 0.90)* |
| Health literacy |  |
| Low | 0.76 (0.75, 0.77)* |
| **Path 3. Lifestyle, social, and psychological factors and MetS** |  |
| **Lifestyle factors** |  |
| Occupational moderate-to-vigorous physical activity |  |
| No | 1.04 (0.96, 1.12) |
| Leisure time moderate-to-vigorous physical activity |  |
| No | 1.29 (1.22, 1.37)* |
| Smoking |  |
| Former | 1.12 (1.05, 1.19)* |
| Current | 1.46 (1.36, 1.56)* |
| Alcohol intake |  |
| No | 1.44 (1.34, 1.55)* |
| Excessive | 1.05 (0.98, 1.12) |
| Diet quality |  |
| Moderate | 1.21 (1.08, 1.35)* |
| Poor | 1.37 (1.19, 1.57)* |
| Sleep duration |  |
| <7 | 1.17 (1.08, 1.26)* |
| >9 | 1.41 (1.12, 1.78)* |
| **Social factors** |  |
| Network size |  |
| <5 | 1.07 (0.97, 1.19) |
| 5-9 | 0.98 (0.90, 1.07) |
| >=15 | 1.10 (1.02, 1.18)* |
| Quality of social contacts |  |
| <=14 | 1.02 (0.96, 1.10) |
| 15-17 | 0.96 (0.90, 1.02) |
| Partner status |  |
| No | 1.21 (1.12, 1.31)* |
| **Psychological factors** |  |
| Self-management skills |  |
| <=66 | 1.09 (1.03, 1.17)* |
| Health literacy |  |
| Low | 1.12 (1.04, 1.20)* |
| **Path 4. Education and MetS controlled for lifestyle, social, and psychological factors** | 0.92 (0.91, 0.94)* |
| Lifestyle factors | 0.92 (0.91, 0.93)* |
| Social factors | 0.90 (0.89, 0.91)* |
| Psychological factors | 0.91 (0.90, 0.92)* |

OR: odds ratio; CI: confidence interval; MetS: metabolic syndrome; the analyses were controlled for age and sex at baseline and time between baseline and the second assessment; reference categories for the lifestyle factors were occupational physical active, leisure time physical active, never smoker, moderate alcohol intake, high diet quality and 7-9 hours of sleep, reference categories for the social factors were a network of 10-14 persons, ≥18 points on the Social Production Function Instrument and having a partner, and reference categories for the psychological factors were 67-100 on the Self-Management Ability Score and 13-15 points on the Brief Health Literacy Screening; * P<0.05.

**Table S6.** Multivariable mediation analysis of lifestyle, social, and psychological factors in the associations between education and incident metabolic syndrome using the Karlson-Holm-Breen method (n = 79,829; 60.7% Female).

|  | **OR (95% CI)** |
| --- | --- |
| **Lifestyle factors** |  |
| Total association | 0.90 (0.89, 0.91)* |
| Direct association | 0.92 (0.91, 0.93)* |
| Indirect association | 0.98 (0.98, 0.98)* |
| Mediating effects (%) |  |
| Total | 19.7 |
| Occupational moderate-to-vigorous physical activity | -1.1 |
| Leisure time moderate-to-vigorous physical activity | 2.6 |
| Smoking | 8.4 |
| Alcohol intake | 5.2 |
| Diet quality | 3.8 |
| Sleep duration | 0.8 |
|  |  |
| **Social factors** |  |
| Total association | 0.90 (0.89, 0.91)* |
| Direct association | 0.90 (0.89, 0.91)* |
| Indirect association | 1.00 (1.00, 1.00)* |
| Total | 1.8 |
| Network size | -0.2 |
| Quality of social contacts | 1.0 |
| Partner position | 1.1 |
|  |  |
| **Psychological factors** |  |
| Total association | 0.90 (0.89, 0.91)* |
| Direct association | 0.91 (0.90, 0.92)* |
| Indirect association | 0.99 (0.99, 1.00)* |
| Total | 7.5 |
| Self-management skills | 2.3 |
| Health literacy | 5.3 |

OR: odds ratio; CI: confidence interval; MetS: metabolic syndrome; the analyses were controlled for age and sex at baseline and time between baseline and the second assessment; * P<0.05.

**Table S7.** Multivariable logistic regression analysis of the direct associations between education, lifestyle, social, and psychological factors and having recovered from metabolic syndrome (n = 13,420; 48.0% Female).

|  | **OR (95% CI)** |
| --- | --- |
| **Path 1. Education and MetS** | 1.05 (1.05, 1.06)* |
| **Path 2. Education and lifestyle, social, and psychological factors** |  |
| **Lifestyle factors** |  |
| Occupational moderate-to-vigorous physical activity |  |
| No | 1.18 (1.16, 1.21)* |
| Leisure time moderate-to-vigorous physical activity |  |
| No | 0.95 (0.94, 0.97)* |
| Smoking |  |
| Former | 0.98 (0.96, 1.00)* |
| Current | 0.91 (0.89, 0.93)* |
| Alcohol intake |  |
| No | 0.88 (0.86, 0.90)* |
| Excessive | 0.97 (0.95, 0.99) |
| Diet quality |  |
| Moderate | 0.92 (0.90, 0.95)* |
| Poor | 0.83 (0.80, 0.86)* |
| Sleep duration |  |
| <7 | 1.00 (0.98, 1.02) |
| >9 | 0.89 (0.84, 0.94) |
| **Social factors** |  |
| Network size |  |
| <5 | 0.89 (0.87, 0.92)* |
| 5-9 | 0.99 (0.97, 1.01) |
| >=15 | 1.03 (1.01, 1.05)* |
| Quality of social contacts |  |
| <=14 | 0.89 (0.87, 0.90)* |
| 15-17 | 1.00 (0.98, 1.01) |
| Partner status |  |
| No | 0.98 (0.95, 1.00)* |
| **Psychological factors** |  |
| Self-management skills |  |
| <=66 | 0.89 (0.88, 0.91)* |
| Health literacy |  |
| Low | 0.76 (0.74, 0.77)* |
| **Path 3. lifestyle, social, and psychological factors and MetS** |  |
| Lifestyle factors |  |
| Occupational moderate-to-vigorous physical activity |  |
| No | 1.03 (0.94, 1.14) |
| Leisure time moderate-to-vigorous physical activity |  |
| No | 0.87 (0.81, 0.93)* |
| Smoking |  |
| Former | 1.00 (0.92, 1.08) |
| Current | 0.87 (0.79, 0.95)* |
| Alcohol intake |  |
| No | 0.86 (0.78, 0.94)* |
| Excessive | 1.05 (0.97, 1.14) |
| Diet quality |  |
| Moderate | 0.94 (0.82, 1.08) |
| Poor | 0.74 (0.61, 0.89)* |
| Sleep duration |  |
| <7 | 0.82 (0.75, 0.91)* |
| >9 | 1.09 (0.79, 1.50) |
| **Social factors** |  |
| Network size |  |
| <5 | 1.19 (1.05, 1.35)* |
| 5-9 | 1.02 (0.91, 1.14) |
| >=15 | 1.04 (0.95, 1.14) |
| Quality of social contacts |  |
| <=14 | 0.99 (0.91, 1.08) |
| 15-17 | 1.01 (0.93, 1.10) |
| Partner status |  |
| No | 0.77 (0.69, 0.86)* |
| **Psychological factors** |  |
| Self-management skills |  |
| <=66 | 0.89 (0.82, 0.96)* |
| Health literacy |  |
| Low | 1.00 (0.92, 1.09) |
| **Path 4. Education and MetS controlled for lifestyle, social, and psychological factors** | 1.04 (1.02, 1.06)* |
| Lifestyle factors | 1.04 (1.02, 1.06)* |
| Social factors | 1.05 (1.03, 1.07)* |
| Psychological factors | 1.05 (1.03, 1.06)* |

OR: odds ratio; CI: confidence interval; MetS: metabolic syndrome; the analyses were controlled for age and sex at baseline and time between baseline and the second assessment; reference categories for the lifestyle factors were occupational physical active, leisure time physical active, never smoker, moderate alcohol intake, high diet quality and 7-9 hours of sleep, reference categories for the social factors were a network of 10-14 persons, ≥18 points on the Social Production Function Instrument and having a partner, and reference categories for the psychological factors were 67-100 on the Self-Management Ability Score and 13-15 points on the Brief Health Literacy Screening; * P<0.05.

**Table S8.** Multivariable mediation analysis of lifestyle, social, and psychological factors in the associations between education and having recovered from metabolic syndrome using the Karlson-Holm-Breen method (n = 13,420; 48.0% Female).

|  | **OR (95% CI)** |
| --- | --- |
| **Lifestyle factors** |  |
| Total association | 1.05 (1.03, 1.07)* |
| Direct association | 1.04 (1.02, 1.06)* |
| Indirect association | 1.01 (1.01, 1.01)* |
| Mediating effects (%) |  |
| Total | 20.6 |
| Occupational moderate-to-vigorous physical activity | 1.5 |
| Leisure time moderate-to-vigorous physical activity | 3.3 |
| Smoking | 4.3 |
| Alcohol intake | 5.7 |
| Diet quality | 6.1 |
| Sleep duration | -0.3 |
|  |  |
| **Social factors** |  |
| Total association | 1.05 (1.03, 1.07)* |
| Direct association | 1.05 (1.03, 1.07)* |
| Indirect association | 1.00 (1.00, 1.00) |
| Total | -1.5 |
| Network size | -3.7 |
| Quality of social contacts | 0.9 |
| Partner status | 1.3 |
|  |  |
| **Psychological factors** |  |
| Total association | 1.05 (1.03, 1.06)* |
| Direct association | 1.05 (1.03, 1.06)* |
| Indirect association | 1.00 (1.00, 1.01) |
| Total | 6.6 |
| Self-management skills | 6.4 |
| Health literacy | 0.2 |

| OR: odds ratio; CI: confidence interval; MetS: metabolic syndrome; the analyses were controlled for age and sex at baseline and time between baseline and the second assessment; * P<0.05. |
| --- |

**Table S9.** Multivariable logistic regression analysis of the direct associations between education and the most important mediating factors and incident and having recovered from metabolic syndrome (n = 93,249; 58.9% Female).

|  | **MetS incidence** | **MetS remission** |
| --- | --- | --- |
|  | **OR (95% CI)** | **OR (95% CI)** |
| **Path 1. Education and outcome** | 0.90 (0.89, 0.91)* | 1.05 (1.03, 1.06)* |
| **Path 2. Education and most important mediating factors** |  |  |
| Smoking |  |  |
| Former | 0.93 (0.92, 0.94)* | 0.98 (0.96, 1.00)* |
| Current | 0.84 (0.84, 0.85)* | 0.91 (0.89, 0.93)* |
| Alcohol intake |  |  |
| No | 0.88 (0.88, 0.89)* | 0.88 (0.86, 0.90)* |
| Excessive | 0.96 (0.95, 0.97)* | 0.97 (0.95, 0.99)* |
| Diet quality |  |  |
| Low | 0.73 (0.72, 0.74)* | 0.83 (0.80, 0.86)* |
| Moderate | 0.86 (0.85, 0.87)* | 0.92 (0.90, 0.95)* |
| Health literacy |  |  |
| Low | 0.76 (0.75, 0.77)* | 0.76 (0.74, 0.77)* |
| **Path 3. Most important mediating factors and outcome** |  |  |
| Smoking |  |  |
| Former | 1.12 (1.05, 1.20)* | 0.99 (0.91, 1.08) |
| Current | 1.50 (1.40, 1.61)* | 0.85 (0.77, 0.94)* |
| Alcohol intake |  |  |
| No | 1.46 (1.36, 1.58)* | 0.86 (0.78, 0.94)* |
| Excessive | 1.04 (0.98, 1.11) | 1.06 (0.97, 1.15) |
| Diet quality |  |  |
| Low | 1.43 (1.25, 1.65)* | 0.71 (0.59, 0.86) |
| Moderate | 1.24 (1.11, 1.38)* | 0.92 (0.80, 1.06) |
| Health literacy |  |  |
| Low | 1.11 (1.04, 1.19)* | 0.99 (0.91, 1.08) |
| **Path 4. Education and outcome controlled for most important mediating factors** | 0.92 (0.91, 0.93)* | 1.04 (1.02, 1.06)* |

OR: odds ratio; CI: confidence interval; MetS: metabolic syndrome; the analyses were controlled for age and sex at baseline and time between baseline and the second assessment; reference categories were never smoker, moderate alcohol intake, ≥18 points on the Social Production Function Instrument and 13-15 points on the Brief Health Literacy Screening; * P<0.05.

**Table S10.** Multivariable mediation analysis of the most important mediating factors in the association between education and incident and having recovered from metabolic syndrome using the Karlson-Holm-Breen method (n = 93,249; 58.9% Female).

|  | **MetS incidence** | **MetS remission** |
| --- | --- | --- |
|  | **OR (95% CI)** | **OR (95% CI)** |
| Total association | 0.90 (0.89, 0.91)* | 1.05 (1.03, 1.07)* |
| Direct association | 0.92 (0.91, 0.93)* | 1.04 (1.02, 1.06)* |
| Indirect association | 0.98 (0.97, 0.98)* | 1.01 (1.00, 1.01)* |
| Mediating effects (%) |  |  |
| Total | 23.9 | 18.0 |
| Smoking | 9.0 | 4.9 |
| Alcohol intake | 5.4 | 5.7 |
| Diet quality | 4.4 | 6.7 |
| Health literacy | 5.0 | 0.6 |

OR: odds ratio; CI: confidence interval; MetS: metabolic syndrome; the analyses were controlled for age and sex at baseline and time between baseline and the second assessment; * P<0.05.

**Table S11.** Educational differences in the development of MetS over the life course stratified by sex, estimated based on simulation.

|  |  | Life course prevalence | | Mean age of onset | | Mean duration | |
| --- | --- | --- | --- | --- | --- | --- | --- |
| Sex | Simulation* | Educational difference^a^  (% points) | Change in the counterfactual (%^b^) | Educational difference^a^ (years) | Change in the counterfactual (%^b^) | Educational difference^a^ (years) | Change in the counterfactual (%^b^) |
| Females |  |  |  |  |  |  |  |
|  | Counterfactual joint effect | 22.015 | 17.8 | 1.506 | 21.2 | 1.903 | 19.0 |
|  | Counterfactual smoking behaviour | 24.645 | 7.9 | 1.723 | 9.8 | 2.162 | 7.9 |
|  | Counterfactual alcohol intake | 25.531 | 4.6 | 1.795 | 6.1 | 2.180 | 7.2 |
|  | Counterfactual health literacy | 25.997 | 2.9 | 1.827 | 4.4 | 2.244 | 4.5 |
|  | Counterfactual diet quality | 26.177 | 2.2 | 1.851 | 3.1 | 2.273 | 3.2 |
|  | Observed | 26.771 | - | 1.910 | - | 2.349 | - |
| Males |  |  |  |  |  |  |  |
|  | Counterfactual joint effect | 23.663 | 16.2 | 2.154 | 20.5 | 2.335 | 18.8 |
|  | Counterfactual smoking behaviour | 26.232 | 7.1 | 2.459 | 9.2 | 2.706 | 5.9 |
|  | Counterfactual alcohol intake | 27.053 | 4.2 | 2.557 | 5.6 | 2.720 | 5.4 |
|  | Counterfactual health literacy | 27.515 | 2.6 | 2.606 | 3.8 | 2.830 | 1.6 |
|  | Counterfactual diet quality | 27.674 | 2.0 | 2.633 | 2.8 | 2.849 | 0.9 |
|  | Observed | 28.243 | - | 2.709 | - | 2.876 | - |

*Simulation: The transition rates for the observed data simulation are based on the values seen in the Lifelines data. The incidence rates for the simulation were estimated using 79,829 individuals (60.7% Female), and the recovery rates were estimated using 13,420 individuals (48.0% Female). The transition rates for the counterfactual simulations assume that the distribution of the modifiable factors for individuals with low education to be the same as individuals with high education. The joint effect represents the situation in which all of the modifiable risk factors were changed.

^a^ The absolute value of the difference between individuals with low and high education

^b^ Percentage change from the observed simulation. Calculated by subtracting the difference between individuals with low and high education in the observed simulation from the difference seen in the counterfactual simulation and dividing by the difference in the observed simulation. Due to rounding, the percentages may differ from the value calculated using the differences presented in the table.

**Table S12.** Educational differences in the development of MetS over the life course stratified by sex: sensitivity analysis.

|  |  | Life course prevalence | | Mean age of onset | | Mean duration | |
| --- | --- | --- | --- | --- | --- | --- | --- |
| Sex | Simulation* | Educational difference^a^  (% points) | Change in the counterfactual (%^b^) | Educational difference^a^ (years) | Change in the counterfactual (%^b^) | Educational difference^a^ (years) | Change in the counterfactual (%^b^) |
| Females |  |  |  |  |  |  |  |
|  | Counterfactual joint effect | 21.574 | 18.1 | 1.384 | 21.7 | 1.863 | 18.7 |
|  | Counterfactual smoking behaviour | 24.210 | 8.1 | 1.593 | 9.8 | 2.114 | 7.8 |
|  | Counterfactual alcohol intake | 25.063 | 4.8 | 1.658 | 6.2 | 2.131 | 7.0 |
|  | Counterfactual health literacy | 25.529 | 3.1 | 1.692 | 4.2 | 2.195 | 4.3 |
|  | Counterfactual diet quality | 25.771 | 2.2 | 1.703 | 3.7 | 2.213 | 3.5 |
|  | Observed | 26.338 | - | 1.767 | - | 2.293 | - |
| Males |  |  |  |  |  |  |  |
|  | Counterfactual joint effect | 23.569 | 16.6 | 2.031 | 19.1 | 2.281 | 18.5 |
|  | Counterfactual smoking behaviour | 26.266 | 7.1 | 2.296 | 8.6 | 2.633 | 5.9 |
|  | Counterfactual alcohol intake | 27.067 | 4.3 | 2.376 | 5.4 | 2.646 | 5.4 |
|  | Counterfactual health literacy | 27.538 | 2.6 | 2.430 | 3.2 | 2.757 | 1.5 |
|  | Counterfactual diet quality | 27.701 | 2.0 | 2.457 | 2.2 | 2.774 | 0.9 |
|  | Observed | 28.276 | - | 2.511 | - | 2.798 | - |

*Simulation: The transition rates for the observed data simulation are based on the values seen in the Lifelines data. The incidence rates for the simulation were estimated using 79,829 individuals (60.7% Female), and the recovery rates were estimated using 13,420 individuals (48.0% Female). The transition rates for the counterfactual simulations assume that the distribution of the modifiable factors for individuals with low education to be the same as individuals with high education. The joint effect represents the situation in which all of the modifiable risk factors were changed.

^a^ The absolute value of the difference between individuals with low and high education

^b^ Percentage change from the observed simulation. Calculated by subtracting the difference between individuals with low and high education in the observed simulation from the difference seen in the counterfactual simulation and dividing by the difference in the observed simulation. Due to rounding, the percentages may differ from the value calculated using the differences presented in the table.

**4. Supplementary Figures.**

**Figure S1.** Monte Carlo variance associated with the estimates of the life course prevalence of MetS.

*****The simulations were repeated 20 times per sample size, and the standard deviation of the parameter was then estimated per sex- and education groups.

^¶^Note: The incidence rates for the simulation were estimated using 79,829 individuals (60.7% Female), and the recovery rates were estimated using 13,420 individuals (48.0% Female).

**Figure S2.** Monte Carlo variance associated with the estimates of the mean age of onset of MetS.

*****The simulations were repeated 20 times per sample size, and the standard deviation of the parameter was then estimated per sex- and education groups.

^¶^Note: The incidence rates for the simulation were estimated using 79,829 individuals (60.7% Female), and the recovery rates were estimated using 13,420 individuals (48.0% Female).

**Figure S3.** Monte Carlo variance associated with the estimates of the mean duration of MetS.

*****The simulations were repeated 20 times per sample size, and the standard deviation of the parameter was then estimated per sex- and education groups.

^¶^Note: The incidence rates for the simulation were estimated using 79,829 individuals (60.7% Female), and the recovery rates were estimated using 13,420 individuals (48.0% Female).

**Figure S4.** Flowchart of the population with and without MetS at baseline.


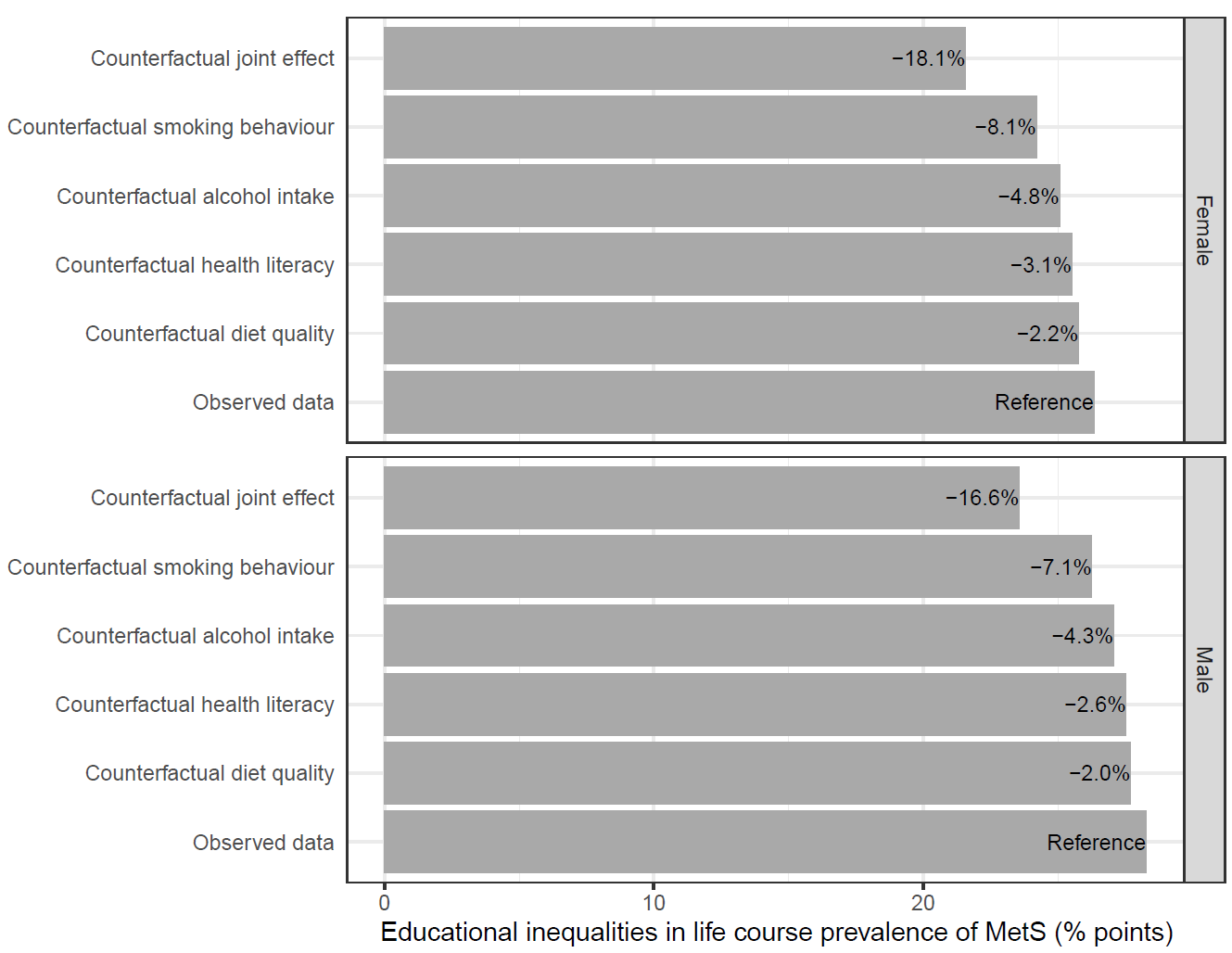


**Figure S5.** Potential impact of modifiable factors on educational inequalities in life course prevalence of MetS between ages 18 and 65. Sensitivity analysis in which transitions with a duration of less than six months were discarded. Results presented separately for each simulation.

*Note: The bars show the additional proportion of individuals with low education who ever experience MetS compared to individuals with high education under different counterfactual conditions. The percentages shown on the bars represent the percentage reduction under the given counterfactual. The incidence rates for the simulation were estimated using 79,829 individuals (60.7% Female), and the recovery rates were estimated using 13,420 individuals (48.0% Female).


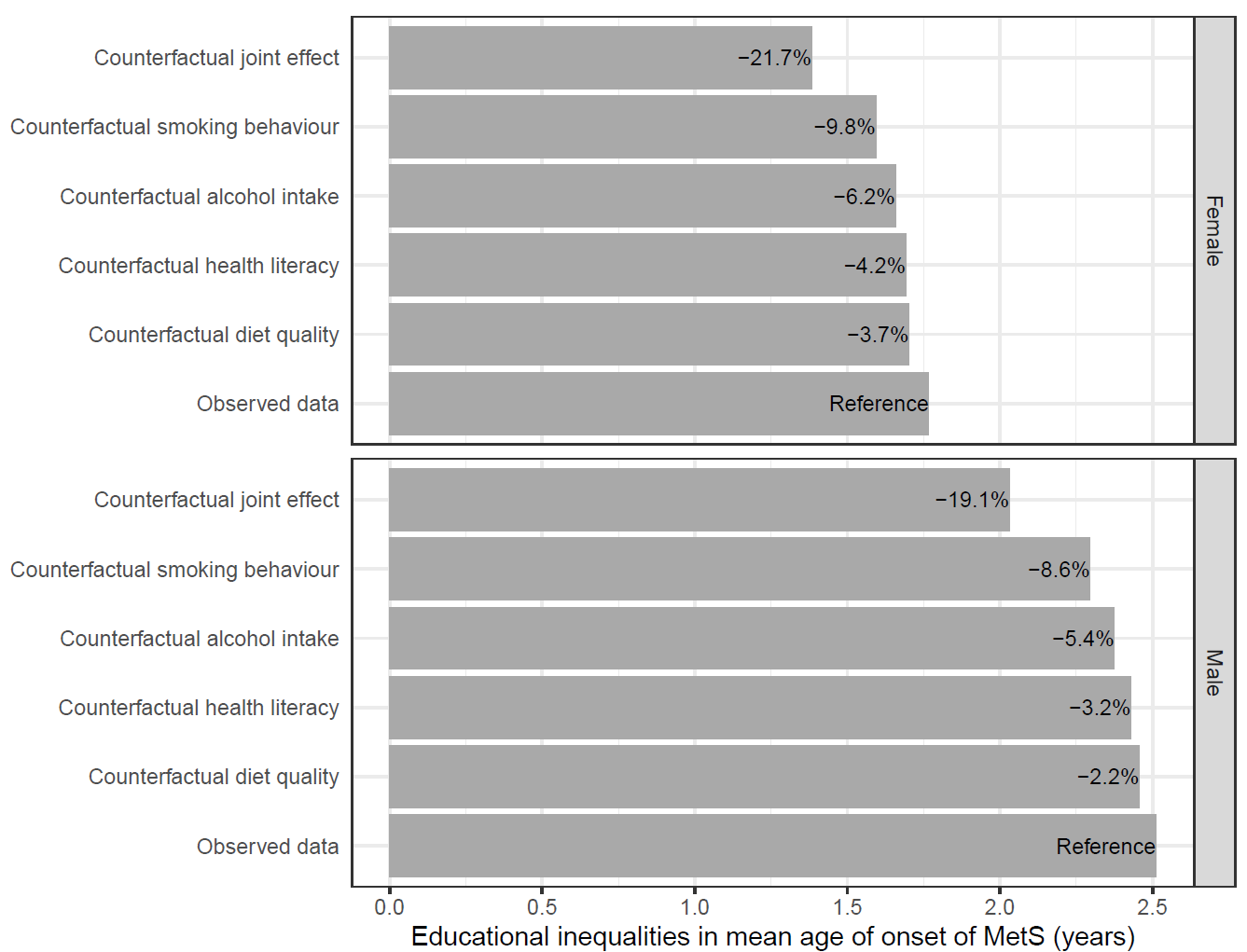


**Figure S6.** Potential impact of modifiable factors on educational inequalities in mean age of onset of MetS between ages 18 and 65. Sensitivity analysis in which transitions with a duration of less than six months were discarded. Results presented separately for each simulation.

*Note: The bars show the difference in the mean age of onset between individuals with low and high education under different counterfactual conditions. Larger values indicate earlier age of onset amongst individuals with low education. The percentages shown on the bars represent the percentage reduction under the given counterfactual. The incidence rates for the simulation were estimated using 79,829 individuals (60.7% Female), and the recovery rates were estimated using 13,420 individuals (48.0% Female).


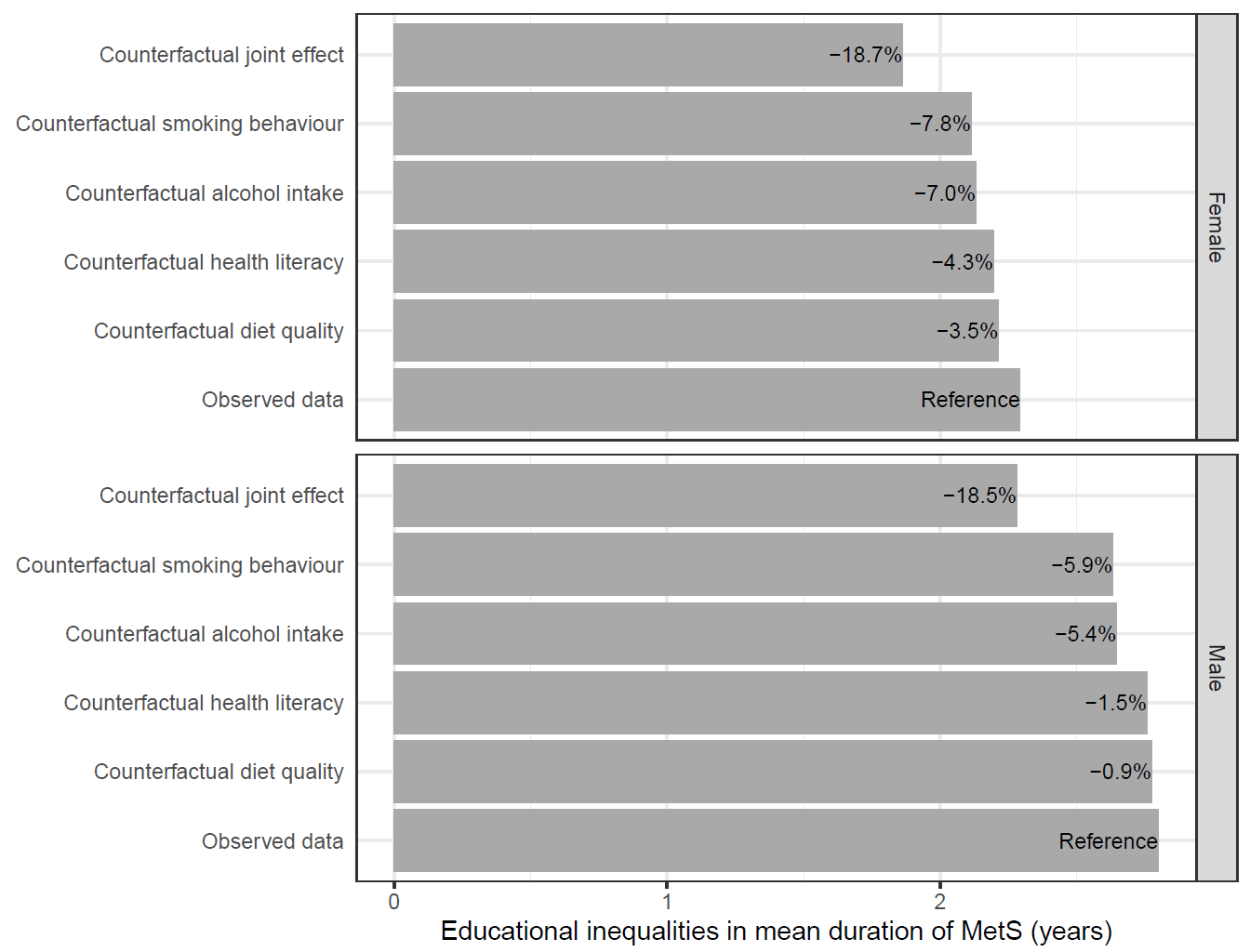


**Figure S7.** Potential impact of modifiable factors on educational inequalities in mean duration of MetS between ages 18 and 65. Sensitivity analysis in which rates for recurrence of MetS are derived using a sample with a history of depression.

*Note: The bars show the additional number of years spent with MetS for individuals with low education compared to individuals with high education under different counterfactual conditions. The percentages shown on the bars represent the percentage reduction under the given counterfactual. The incidence rates for the simulation were estimated using 79,829 individuals (60.7% Female), and the recovery rates were estimated using 13,420 individuals (48.0% Female).

**Figure S8.** Observed (points) and expected (lines) values of transition rates for males and females by education level.

*Note: The incidence rates for the simulation were estimated using 79,829 individuals (60.7% Female), and the recovery rates were estimated using 13,420 individuals (48.0% Female).
